# Tele-Monitoring Applications in Respiratory Allergy

**DOI:** 10.3390/jcm13030898

**Published:** 2024-02-04

**Authors:** Xenofon Aggelidis, Maria Kritikou, Michael Makris, Michael Miligkos, Niki Papapostolou, Nikolaos G. Papadopoulos, Paraskevi Xepapadaki

**Affiliations:** 1 Allergy Unit, 2nd Department of Dermatology and Venereology, Medical School, Attikon University Hospital, National and Kapodistrian University of Athens, 15772 Athens, Greece; zenossag@yahoo.gr (X.A.); mmakris.allergy@gmail.com (M.M.); nikipapap@gmail.com (N.P.); 2 Allergy Department, 2nd Pediatric Clinic, National and Kapodistrian University of Athens, 15772 Athens, Greece; mmiligkos@med.uoa.gr (M.M.); nikpap@med.uoa.gr (N.G.P.); vxepapadaki@med.uoa.gr (P.X.)

**Keywords:** respiratory allergy, tele-monitoring, adherence, telemedicine, asthma, applications

## Abstract

Respiratory allergic diseases affect over 500 million people globally and pose a substantial burden in terms of morbidity, mortality, and healthcare costs. Restrictive factors such as geographical disparities, infectious pandemics, limitations in resources, and shortages of allergy specialists in underserved areas impede effective management. Telemedicine encompasses real-time visits, store-and-forward option triage, and computer-based technologies for establishing efficient doctor-patient communication. Recent advances in digital technology, including designated applications, informative materials, digital examination devices, wearables, digital inhalers, and integrated platforms, facilitate personalized and evidence-based care delivery. The integration of telemonitoring in respiratory allergy care has shown beneficial effects on disease control, adherence, and quality of life. While the COVID-19 pandemic accelerated the adoption of telemedicine, certain concerns regarding technical requirements, platform quality, safety, reimbursement, and regulatory considerations remain unresolved. The integration of artificial intelligence (AI) in telemonitoring applications holds promise for data analysis, pattern recognition, and personalized treatment plans. Striking the balance between AI-enabled insights and human expertise is crucial for optimizing the benefits of telemonitoring. While telemonitoring exhibits potential for enhancing patient care and healthcare delivery, critical considerations have to be addressed in order to ensure the successful integration of telemonitoring into the healthcare landscape.

## 1. Introduction

Respiratory allergic diseases, including rhinitis and asthma, affect more than 500 million people worldwide and impose an enormous morbidity and economic burden, attributed to either direct or indirect healthcare costs, i.e., lost days at work or school. Despite significant improvements in our knowledge, asthma and rhinitis management remain challenging [1]. Effective management encompasses several domains, such as education of health providers and patients, environmental control, frequent medical consultations for assessing adherence, follow-up, and management, ideally by a respiratory physician specialist. Nevertheless, several constraints, such as geographical disparities in access, infectious pandemics, a lack of economic resources, the shortage of allergy specialists in rural and underserved urban communities, and the well-acknowledged suboptimal adherence, halt the proper management of allergic patients.

Telemedicine (TM) modes for asthma care include synchronous, i.e., “real-time patient visits”, asynchronous options based on "store-and-forward”, and triage. TM deploys computer-based technologies to achieve efficient doctor-patient communication and facilitate distant health care encounters [2]. Although TM indices were used for years, it was only during the COVID pandemic that such processes were implemented as mainstream clinical practice and the start-up point to set up an infrastructure for providing care in the forthcoming years in allergy practice [3]. Nevertheless, as emerging technologies are massively increasing, certain concerns regarding the quality of different TM platforms, safety, parity reimbursement, doctor and patient satisfaction, and effectiveness on clinically relevant disease outcomes are scrutinized [4]. Of importance, regulatory, legal, and ethical considerations with respect to TM elements are constantly reviewed by the respective authorities worldwide.

Recent advances in digital technology for monitoring and improving treatment adherence in individuals with asthma and rhinitis across all age groups include specifically designated applications, informative material, digital examination devices such as stethoscopes, high-resolution cameras, audio conferences, intergraded platforms, and electronic medical files [5]. In parallel, employing appropriate assessment tools for evaluating the different aspects of TM systems is needed [1]. The present review will outline the advances in TM information, communication, and applications in respiratory allergy, analyze the current shortcomings and concerns regarding their implementation in the different age groups, and discuss privacy and insurance issues and their translation into clinically meaningful disease outcomes.

## 2. Epidemiology and Economic Burden of Allergic Rhinitis and Asthma

The upper and lower airways were traditionally considered separate systems with distinct pathologies, thus limiting the full elucidation of their function. Nevertheless, this notion has been totally revised by the Allergic Initiative and its impact on Asthma (ARIA) consortium during the last two decades [6].

Epidemiological data on the prevalence of asthma worldwide are challenging to estimate due to differences in the survey, diagnostic, and reporting methods. The International Study of Asthma and Allergies in Children (ISAAC) showed wide variations in asthma prevalence within the twenty-one participating centers, with rates ranging from 5 to over 20% in the general population, while a northwest to southeast gradient was noted, more so in the eastern continent [7]. Asthma prevalence in Europe, as presented in the European Lung White book, ranges from 1.4% in Bosnia-Herzegovina to 20.6% in Sweden [8], while in Asia and America, it ranges from 1.3% in Nepal to 13.0% in Peru, respectively [9]. In the systematic analysis of the Global Burden of Disease Initiative 2015, it was estimated that approximately 358 million individuals have asthma [10]. More recently, the Global Asthma Network (GAN) Phase I study, using the same questionnaires as in the ISAAC study, showed that the overall prevalence of current asthma symptoms between 2015 and 2020 was 9.1% for children, 11.0% for adolescents, and 6.6% for adults, with wide variations between centers, both within and between countries [11].

The prevalence of allergic rhinitis (AR) among 6- to 7-year-old children ranged from 6.4% in Eastern and Northern Europe to 7.3% in Western Europe in the ISAAC study, while increased prevalence rates were reported in the 13–14-year-old group, ranging from 10.5% in Eastern and Northern Europe to 14.5% in Western Europe [7]. In European adults, the estimated overall prevalence, as assessed by the ARIA definition for allergic rhinitis, was 25%, ranging from 17% in Italy to 28.5% in Belgium, thus affecting almost 500 million people around the world [12,13].

Both asthma and allergic rhinitis impose a substantial health loss burden, both at an individual and societal level. The costs are analyzed as either direct, including the formal health care services, or indirect, as assessed by absenteeism and presentism. Accordini et al. estimated the total asthma cost in Europe at €19.3 billion, with 62.5% attributed to indirect non-medical costs [14]. Although direct comparisons are difficult to perform between countries, a study evaluating indirect costs has shown an almost 10-fold variation among different countries, with the lowest note in the Republic of Korea ($142/per year) and the highest in the U.S. ($1274/per year) [15].

Although the estimated direct costs for AR per patient per year are lower than those with asthma, the higher prevalence of AR and the usually concomitant asthma comorbidity increase the total economic cost [16].

## 3. Toward a Personalized Approach to Managing Patients with Respiratory Allergies

Despite our advanced understanding with respect to the pathophysiological mechanisms underlying asthma and AR, disease control remains suboptimal in a significant proportion of patients. The evolution of phenotypes and respective endotypes in respiratory allergic diseases, the introduction of invasive and non-invasive respective biomarkers, and the advent of monoclonal antibodies for severe asthma patients have significantly contributed to and advanced the concept of a precision treatment approach [11]. In this context, the evidence-based guidelines (ARIA) for AR patients suggest a stepwise diagnostic and treatment pathway, depending on the AR severity and duration [13,17]. With respect to asthma patients, the outdated “one-size-fits-all” therapeutic approach has been totally revised, allowing for more targeted, according to the individual’s treatable traits, management, as suggested by the Global Initiative for Asthma (GINA) 2023 report, the PRACTALL, and the EUFOREA-ARIA-EPOS-AIRWAYS ICP statements [18,19].

Prior to “the digital age”, respiratory diseases’ monitoring was mainly performed during patients’ visits in the clinic or in the doctor’s office. Although essential, objective monitoring tools such as validated questionnaires, lung functional tests, and airway inflammation measurements, such as fractional exhaled nitric oxide_(FeNO), are occasionally performed, thus partially reflecting the overall disease severity and control [20]. Although important, face-to-face consultations are time-limited, costly, and time-consuming, while busy schedules may halt the physician from fully apprehending the impact of illness on patients’ daily lives or inadequately reviewing patients’ education and self-commitment—a common cause of poor treatment adherence [21]. It is well accepted that frequent, according to disease burden, monitoring of allergic patients is essential for early detection of the disease’s loss of control and estimation of an increased exacerbation risk, while expedited urgent consultation and treatment adjustment can reduce hospitalization’s risk and disease morbidity overall and improve quality of life [22].

Nowadays, patient-reported outcome measures (PROMs), although mainly reflecting patients’ perspectives, are considered essential for proper monitoring; thus, they are endorsed by all recent guidelines and major health organizations [23]. Nevertheless, hard-copy questionnaires are infrequently completed, more so on a long-term follow-up basis [24]. Stakeholders state the unmet need for accessible, reliable, noninvasive, and individualized disease monitoring tools as a high priority for children with asthma [25,26]. Moreover, the need for near-real-time and objective measurements to identify potential triggers, including air pollutants, allergens, and tobacco smoke, and to assess physical activity and the actual burden of the disease is also awaited. From the patient’s perspective, access to valid medical information and educational material, reminders to receive treatment, and alerts of imminent disease exacerbations are of importance [27].

E-health strategies aim at overcoming barriers to accessing healthcare services in certain aspects currently hampering optimal disease management, reducing health-related costs and medical errors, and most importantly, encouraging disease awareness and shared decision-making [28,29].

## 4. The Aims of Tele-Monitoring in Managing Patients with Chronic Diseases

The term TM refers to a set of services that are developed on an advanced telecommunications infrastructure and supported by informative technology and related applications. There are terminologies that are closely associated with TM, either as provisions or as tools [30,31,32]. In specific, terms such as telehealth, electronic health, mobile health (mHealth), software applications, etc., refer to clinical or non-clinical services and means used for healthcare delivery, education, monitoring, and assessment of patients/users’ medical conditions. TM encompasses both diagnostics, treatment approaches, and rehabilitation [33,34,35,36]. Detailed descriptions and definitions of distinct telemonitoring features such as telehealth, tele-diagnosis, tele-screening, m-health, e-health, etc., pertaining to the telemedicine domain are now provided by the EAACI position paper dedicated to TM with a special focus on allergic diseases and asthma [36].

The primary aim of TM is not to substitute in-person consultations but rather to serve as a complementary or alternatively effective option for patient monitoring [37]. In this context, TM aims at alleviating the overcrowded primary healthcare system by providing uninterrupted access to services for all, especially the socioeconomically disabled and remote areas’ residents, and enhancing disease control. TM addresses the gaps in accessibility, equity, and inequality in health services, as well as cost-disparities [35,38]. It has been mostly implemented in patients with chronic and non-communicable diseases such as diabetes mellitus, rheumatologic diseases, and chronic allergy-associated respiratory diseases such as asthma and allergic rhinitis, where regular monitoring is essential for better disease control [33,38].

Telemonitoring applications have shown beneficial effects by means of reduced medication use and improved adherence in patients with asthma and allergic rhinitis [39,40]. Data from a global study in 2020 highlighted the reduced morbidity of children with asthma during the first COVID pandemic wave, partially attributed to reducing in-person visits by incorporating TM approaches, while adherence was significantly pronounced [41]. Significant advantages have been documented regarding the effectiveness of telemedicine in disease control compared to in-person visits. Additionally, the majority of patients expressed a positive attitude toward telehealth, as indicated by respective satisfaction survey results (~95%) [5]. Recent data are also confirmative of the TMs effectiveness both in disease management and the quality of life of asthmatic patients [22,42,43,44,45,46,47].

Implementation of TM in patients with respiratory allergies should comply with certain descriptives, such as the introduction of a user-friendly, cost-effective, and interactive interface and the incorporation of practices that increase accessibility (such as training and education, privacy measures, support, etc.) by logging in symptoms and medication use and increasing disease awareness both for the health provider and the patient [48,49]. Moreover, app personalization, meaning tailoring the app to the individual’s specific needs and preferences, including customized medication reminders, action plans, and allergen information, is an unmet need.

## 5. Means for Telemonitoring Patients with Respiratory Allergic Diseases

Text messages: Short message service reminders have been utilized in the care of chronic medical diseases in the last two decades [50]. Their utility in improving medication adherence and clinical outcomes in asthma patients has been confirmed in a recent Cochrane systematic review [51]. Short-term reminders resulted in increased adherence, both in adults and in children, whereas improved asthma control was also achieved. However, the exact effect of these reminders on other clinically important outcomes, such as asthma exacerbations or lung function, as well as the long-term effects, remain to be explored.

Games: Serious games are educational tools that have been proposed for promoting self-management in asthma patients. Their interactive, easy-to-use, and rewarding interface renders them particularly appealing to children. A systematic review of 10 serious games used in randomized controlled trials of 3–10-year-old asthmatic children showed significantly increased asthma knowledge, albeit consistent behavioral changes, and clinical outcomes (e.g., emergency department visits, hospital admission, asthma symptoms) were not improved [52]. Involvement of parents or other children as simultaneous players, implications of real-life scenarios, and integration within mobile applications instead of computer-based systems may facilitate the translation of the acquired knowledge into meaningful clinical results [53].

Inhaler device modification, built-in sensors: Digital inhalers were recently introduced and have a continuously growing market share. These include conventional inhalers with add-on sensors, compatible with most powered meter-dose inhalers (pMDIs) and dry power inhalers (DPIs), or “smart” inhalers with embedded sensors. They assess medication adherence by detecting the least time and number of actuations used, while inhalation technique can be recorded in a subset [54]. A recent meta-analysis demonstrated significantly increased adherence in patients with asthma, whereas a marginally beneficial effect on asthma control was shown in a proportion of the studies [51]. In specific, the INhaler Compliance Assessment (INCA) device is an attached sensor that creates audio recordings and, through sound analysis, assesses the inhalation technique and therefore “actual” adherence to the medication [55]. Moreover, the digihaler is an FDA-approved device containing albuterol, fluticasone, or fluticasone-salmeterol with integrated sensors that record peak inspiratory flow (PIF), volume, duration, and time to PIF. In a 3-month randomized controlled trial in adolescents and adults, the use of the albuterol Digihaler resulted in improved inhalation technique and clinically meaningful improvements in asthma control compared to the albuterol inhaler without the digital component [56].

Integrated platforms: The latest innovation in the electronic monitoring of patients with respiratory allergies is the advent of platforms, which integrate several digital components. They typically consist of a “smart” inhaler, which connects to a designated mobile application, a cloud server to store data, and separate dashboards transferring respective data to either patients and/or healthcare professionals. Moreover, they record adherence and inspiratory flow, send inhaler or mobile application alerts, and provide feedback for optimal inhaler technique. The patient’s interface presents relevant, actively or passively acquired data to the user to promote disease self-management, whereas the doctor’s interface facilitates clinical monitoring and detection of actual drug delivery issues. The efficacy of these digital solutions has been explored in a few RCTs [56,57], while several others are currently underway [58]. The positive impact of these platforms on adherence and inhaler technique, as well as on asthma control and rescue medication use, is evident across studies.

Wearables: Wearable devices include smartwatches, bracelets, bands, or rings and allow for the passive, non-intrusive, outpatient, real-time collection of clinical data. They usually record physiological parameters, e.g., vital signs, oxygen saturation, sleep quality, and physical activity measures. In an early pilot study of 22 asthmatic adolescents, the Fitbit^®^-derived quality of sleep and physical activity measures did not match the self-reported quality of sleep and pediatric asthma impact scores, respectively [59]. Nevertheless, a recent study, including machine learning (ML) models combined with relevant telemonitoring data, successfully predicted acute asthma exacerbations in children and adolescents [60]. The integration of the Internet of Things and Artificial Intelligence technologies into everyday clinical practice is expected to translate clinical data into personalized, evidence-based care delivery.

## 6. Assessment Tools for Remote Patient Monitoring

Disease awareness is a key component for the efficient control of chronic respiratory allergies; to achieve this, a series of diagnostic and monitoring devices for home use are commercially available. The currently marked digital stethoscopes can efficiently remove non-essential noise, while they are combined with artificial intelligence (AI) algorithms that recognize, amplify, visualize, and record breath sounds such as wheezing and coughing with substantial sensitivity [61,62]. Patients or caregivers can also utilize portable digital spirometers or peak flow meters for both self-assessment and guided treatment while reviewing effort quality and, in certain cases, in a visualized, easy-to-understand form, their effort results (e.g., traffic light system) [63]. Moreover, current and historical data can be transmitted via a smartphone to the treating physician in an (un)synchronized mode. Efforts are underway to combine multiple digital devices’ data for optimal monitoring of respiratory allergies. In this respect, MyAirCoach is a clinical program that utilizes an inhaler adapter, an indoor air-quality monitor, a physical activity tracker, a portable spirometer, an FeNO meter, and an app, providing a dataset to the treating physician through a web-based platform [64].

Action plans: Patients with asthma and rhinitis are currently encouraged towards self-management under professional supervision, an approach with considerable benefits for achieving disease control [65]. Asthma action plans, as recommended by international guidelines, combined with proper education and regular consultations, minimize asthma symptoms, absenteeism, and emergency room visits [66]. Nevertheless, action plans are sub-optimally implemented into clinical practice by both general practitioners and allergy specialists [67]. In this context, individualized action plans in a mobile (m) app format were more applicable to adolescents with asthma, resulting in improved disease control [68,69].

The integration of action plans in TM empowers patients to proactively manage their disease by combining real-time data, education, and support. MHealth apps provide allergic patients with personalized avoidance strategies, medication adherence reminders, and forecasts for pollen or air pollutants. Based on symptom scores, objective measurements, and the use of rescue medication, patients are alerted when to seek medical advice or adjust control treatment.

Questionnaires: PROMs are valued for their ability to document patients’ perspectives on disease severity and control, medication use, and other disease aspects like productivity and quality of life [24]. E-diaries are currently included in several health-related apps, designed for daily monitoring of respiratory allergies. Although not superior to paper diaries with respect to asthma control [70], mobile apps are appealing to patients due to their engaging interface and ability to send reminders that reinforce adherence [71]. Disease monitoring through digital questionnaires is accepted by patients, especially in cases of communication barriers such as limited-time visits and living in rural areas [72], although adherence still remains suboptimal [73]. E-diaries document symptoms more accurately than retrospective reporting during visits; thus, mobile apps with validated questionnaires, supported by controlled trials, and advocated by health regulatory organizations are recommended.

Applications: Numerous applications are currently available within the context of digital health (encompassing e-health and m-health), which simultaneously collect real-world data for better disease awareness and management in allergic patients. Recent studies have identified over 1500 available applications for patients with allergic rhinitis/sinusitis, and asthma; however, only a small proportion hold published scientific data regarding their efficacy. The MASK-air application, supported by the Phase 3 ARIA (Allergic Rhinitis and its Impact on Asthma) initiative, is a highly effective and validated mobile health application that aims to improve the diagnosis, monitoring, and self-management of patients with allergic rhinitis and asthma (Table 1 and Table 2). MASK Air is currently used by more than 58,000 people worldwide in a patient-centered approach, facilitating shared decision-making [74,75].

Information for patients (educational): Successful patient engagement and shared decision-making require sufficient patients’ health literacy [76]. Medical information may be acquired from reliable websites and health-related apps. The Internet’s revolutionization of all aspects of education and medical information was not an exception. However, guided by their physicians, patients can now access trustworthy websites, participate in conversations with experts, and receive educational material on the correct use of inhalers and effective allergen avoidance. Digital education in asthma has substantially improved disease knowledge, reduced the need for emergency department visits, and increased the number of symptom-free days [77]. When interactive, educational support positively affects adherence to treatment and limits disease burden [78].

**Table 1:** Most important apps for allergic rhinoconjunctivitis [74] and asthma [79].

	Available Languages	Published Validation Studies	
Allergic Rhinitis			
AllergyMonitor	11	7	symptoms and medication diary, adherence to allergen immunotherapy, sharable reports, pollen forecast, access to a data platform by treating physicians
MASK-air	20	16	symptoms and medication diary, sharable reports, pollen forecast
Pollen App *	6	2	symptoms and medication diary, sharable reports, validated aerobiologic data
Asthma			
AsthmaMD *	1	0	symptoms, triggers, medication diary/reminder, sharable symptoms, and PEF reports, action plans
Asthma Storylines *	7	0	symptoms and medication diary/reminder, sharable reports, validated action plans
ASTHMAXcel *	1	7	educational content
eAMS *	1	1	symptoms, triggers, and medication questionnaires, educational content, a clinical decision support system, and action plans

* Available in limited countries/languages.

**Table 2:** List of selected studies per telemonitoring modality with their respective outcomes.

	Studies in Asthma/Allergic Rhinitis	Statistically Significant Outcomes	Non-Statistically Significant Outcomes
Text messages	Chan et al. [51] [SR of 12 RCTs]	Adherence, asthma control	
Games	Drummond et al. [52] [SR of 12 studies]	Asthma knowledge	Behavioral changes and asthma-related clinical outcomes
Inhaler device modification, built-in sensors	Chan et al. [51] [SR of 10 RCTs]	Adherence, asthma control	
	Hoyte et al. [56] [RCT of 333 adults/adolescents]	Asthma control	
Integrated platforms	Moore et al. [57] [RCT of 437 adults]	Adherence	Asthma control, FeNO, PEF
Wearables	Huffaker et al. [60] [Trial of 16 children/adolescents]	Prediction of loss of asthma control	
	Bian et al. [59] [Trial of 22 adolescents]	Association of patient-reported outcomes with Fitbit-derived sleep quality and physical activity measures	
Mobile asthma action plans	Murphy et al. [69] [SR of 4 studies]		Asthma control
Education	Mosnaim G et al. [78] [Scoping review of 6 RCTs and 3 single-arm studies)	Asthma control and self-reported asthma symptoms	
MHealth apps	Sousa-Pinto et al. [74] [Review of 2 RCTs]	Adherence to allergic rhinitis therapy and monitoring	

Abbreviations: FeNO = Forced expiratory Nitric Oxide; PEF = Peak expiratory flow; SR = Systematic Review.

## 7. Addressing Concerns and Barriers to Telemonitoring Implementation

Telemonitoring represents a transformative force in modern healthcare, offering promising opportunities to enhance patient care and optimize healthcare delivery. However, to fully unlock its potential, critical questions should be addressed regarding doctor-patient collaboration, cost-effectiveness, insurance issues, data privacy, the role of AI, potential workforce implications, and the overall readiness of the healthcare system [80].

One of the main TM concerns is the efficacy of the remote doctor-patient mode of communication and vice versa via the TM applications. The traditional doctor-patient relationship, based on face-to-face interactions, is partly redefined by the virtual nature of telemonitoring. According to the Agency of Healthcare Research and Quality guidelines, there are essential elements for establishing an effective and comparative relationship, such as eye contact, voice intonation, respectful touch, body posture and language, professional appearance, sufficient time and conversational behavior, and the use of physical space [81]. Several communication elements can be transferred through the virtual realm of TM for establishing trustful and collaborative interaction; however, this is infeasible for others. Recent studies evaluating patient-physician interactions in terms of trust communication and interaction show highly favorable patients satisfaction with TM visits, especially during the COVID-19 pandemic [82,83]. However, others underpin the patients’ concerns regarding the impact of TM on physicians’ attentiveness, while doctors state that such systems might provide novel insights into patients’ living circumstances [80]. Additional concerns and obstacles are raised when eHealth is implemented in older patients [84], such as insufficient technical literacy and hesitancy in using digital tools, with apprehensions regarding privacy and security. Of note, infrequent face-to-face visits might enhance loneliness and depression feelings in this age group [85,86]. Inversely, post-implementation studies indicate that technology might empower and activate older patients, suggesting benefits for the elderly [86].

Utilization of telehealth systems for the collection, exchange, and transmission of sensitive health data have repeatedly raised challenges and issues in privacy and security aspects. Warranting that patient information is securely handled, stored, and accessed by authorized personnel is of major concern. Additionally, incorporating TM into insurance models and ensuring appropriate coverage necessitates comprehensive policy frameworks to safeguard both patients and providers. In Europe, TM is acknowledged as both a health and an information service. However, the absence of consistent medical liability and regulation prevents the establishment of a unified European framework. Another challenge is the inadequate governmental support for TM innovation, as well as the absence of social security systems to offset the expenses associated with TM [36]. Privacy and security in telehealth/telemonitoring practices are influenced by three main risk factors: environmental factors, such as inadequate privacy in households due to a lack of space for confidential conversations and challenges in sharing sensitive health information remotely; technology factors, including data security concerns, limited internet access, and technological limitations; and operational factors, i.e., encompassing reimbursement, payer denials, technology accessibility, and training/education requirements. There is an unmet need for establishing guidance for governments, policymakers, and healthcare organizations in formulating optimal approaches to ensure privacy and security in telehealth [87].

Regarding insurance plans, electronic databases raise concerns about the potential vulnerabilities associated with data protection. The storage of sensitive information regarding insurance owners in these databases, coupled with their expansive reach, underscores the potential exposure to cybersecurity risks. It is mandatory for insurance providers to prioritize and implement robust cybersecurity measures to safeguard this data. Individuals who entrust insurance companies with their sensitive information may face threats such as cyber-attacks and identity theft. Therefore, ensuring the security of insurance-related data are not only a regulatory requirement but also essential for maintaining the trust and confidence of policyholders in their insurance companies [88].

Currently, there is a limited body of research outlining the medical and economic effectiveness derived from self-management approaches and digital therapeutic solutions. Three recent studies on telemonitoring for Asthma, COPD, and Cystic Fibrosis demonstrated that technology improves the quality of life, increases access to asthma care, and is cost-effective by saving up to €3600 per patient per year [89]. Nevertheless, upfront expenses associated with implementing and maintaining the necessary technology infrastructure should be accounted for. Healthcare institutions and policymakers currently assess whether the long-term benefits outweigh the initial investments and ongoing operational costs, ensuring that telemonitoring is a sustainable and economical solution in the healthcare landscape.

As we march into the era of TM, the input of artificial intelligence (AI) incorporated into the TM applications should be cautiously assessed. AI-powered algorithms have the potential to analyze vast amounts of patient data swiftly and accurately, assisting healthcare professionals in making informed decisions and potentially hampering human involvement. Finding the right balance between AI-enabled insights and human expertise is essential to optimizing the benefits of TM. The implications of AI and human expertise in telemonitoring services are effective for assessing data analysis, disease risk, providing personalized treatment plans, and assessing patients’ adherence to treatment. Although AI can autonomously handle data analysis, the valuable addition of doctors’ expertise and clinical judgment becomes evident. However, such interaction addresses certain challenges in complex clinical decision-making, emotional and empathetic support, addressing ethical dilemmas, considering cultural nuances, and managing interpersonal communications [90]. There are also concerns about a potential increase in the workload of human employees since the massive influx of data might necessitate additional human effort for interpretation and action. Finding ways to streamline processes, leverage AI for routine tasks such as entry, documentation and initial data analysis, appointment scheduling, medication adherence monitoring, and handling routine follow-up appointments, and empower healthcare professionals to focus on critical decision-making, is crucial in addressing this concern and ensuring the scalability of TM [91]. In summary, in the realm of data analysis, the indispensable role of human doctors cannot be overstated. AI streamlines routine tasks and empowers healthcare professionals to focus on critical decision-making and personalized patient care. The synergy between AI-driven data analysis and human interpretation optimizes efficiency and ensures a comprehensive approach to respiratory allergy telemonitoring.

Whether the current state of TM is advanced and reliable enough for widespread adoption and full support within the healthcare system remains a primary concern.

## 8. Conclusions

TM, including several types of services, modes, delivery models, and means, was widely used after the COVID pandemic for patients with allergy-associated respiratory diseases. It ensures a successful therapeutic alliance as a complementary and efficacious alternative communication pathway between health providers and patients. The beneficial effects and facilitation on asthma diagnosis, assessment, monitoring, adherence to treatment, and quality of life, more so in socioeconomically disabled and remote patients, and on direct and indirect costs, have been clearly documented so far. Nevertheless, there are concerns regarding adequate technology requirements, depersonalization of the patient-doctor relationship, data protection and privacy regulations, legally compliant systems, and compensation. While several unmet needs still have to be addressed, TM is nowadays effective, in parallel with face-to-face consultations, healthcare tool.

## Data Availability

Not applicable.
